# A DNA-based approach to infer species diversity of larvae and adults from the white grub genus *Phyllophaga* (Coleoptera: Scarabeidae)

**DOI:** 10.3389/finsc.2024.1465794

**Published:** 2024-11-07

**Authors:** Ariel W. Guzmán-Franco, Maribel Rivero-Borja, Antonio Marín-Jarillo, Fernando Tamayo-Mejía, Nayra Guzmán-Santillán, Tania Guzmán-Santillán

**Affiliations:** ^1^ Posgrado en Fitosanidad-Entomología y Acarología, Colegio de Postgraduados, Municipio de Texcoco, Estado de Mexico, Mexico; ^2^ Programa de Entomología, Campo Experimental Bajío, Instituto Nacional de Investigaciones Forestales, Agricolas y Pecuarias (INIFAP), Celaya, Guanajuato, Mexico; ^3^ Direccion General Agrícola, Secretaria del Campo, Celaya, Gto, Mexico; ^4^ Escuela de Ciencias, Universidad de las Américas Puebla, Puebla, Mexico

**Keywords:** molecular taxonomy, Cytochrome c oxidase subunit 1, Cytochrome b, 28S rDNA, integrative taxonomy

## Abstract

Scarabaeoidea is a diverse and widely distributed insect group; many are agricultural pests including species within the genus *Phyllophaga*. Species diversity studies in this taxonomic group are done mainly using morphological identification. However, despite existing taxonomic keys for adults and larvae, identification may be difficult due to their complex morphology. Molecular taxonomy can increase the value and accuracy of morphological species identification of larvae and adults. To test this, larvae collected from soil close to maize plants were identified using molecular taxonomy, and compared with adults captured using light traps. The larvae (2021) and adults (2022) were sampled on maize at the same locations in central Mexico. Molecular identification was achieved using three regions within the Cytochrome oxidase gene (*cox*), two in the Cytochrome c oxidase subunit 1 (cox1), Cytochrome b (CytB) and 28S rDNA. *Cox* gene information was more useful than nuclear information (28S). Combined morphological and molecular taxonomy of adults distinguished between seven *Phyllophaga* species. Although two closely related species, *P. polyphyla* and *P. ravida*, were distinguished using *cox* gene information, greater resolution was obtained using CytB. All analyses identified cryptic species within *P. vetula*. Species found amongst sampled adults were similar to those found amongst larvae. However, the number of species was greater in adults than in larvae at the same locations. Larval information showed *Phyllophaga* community structure changed over time. Our findings will contribute to a better understanding of *Phyllophaga’s* ecology in maize.

## Introduction

The superfamily Scarabaeoidea is one of the most diverse and widely distributed insect groups (1). Within this superfamily, the family Scarabeidae comprises many species, some of which are agricultural pests (2). The genus *Phyllophaga* is one of the most economically important within Scarabeidae (2, 3), since larvae feed on plant roots; large infestations can cause plant death (4, 5). Therefore, a key component of any control strategy is accurate species identification because different soil dwelling species may be present in soil but not all of them feed on and damage plant roots (5).

Species identification in the genus *Phyllophaga*, as with many insect species, has been done based on morphological attributes (5–7). Taxonomic keys have been developed for adults (e.g. 8, 9) and larvae (e.g. 10–13) of some species of *Phyllophaga*. However, despite advances in taxonomic keys for identification of species in *Phyllophaga*, morphological identification remains difficult due to the complexity of their morphology requiring a great deal of expertise to achieve accurate identification (11).

DNA based identification is widely used and is becoming an increasingly reliable tool for insect identification (14). Mitochondrial DNA (mtDNA) genes are popular for species-level delimitation because of their maternal inheritance, limited recombination, rapid evolution and high resistance to degradation (15). There are several molecular markers used for particular insect taxa. Regions within the mitochondrial Cytochrome oxidase (*cox*) and nuclear genes have proved to be very useful for species separation in Coleoptera, specifically in the family Scarabaeidae (16–23). Within the *cox* gene, various regions have been used successfully for insect identification; some regions have proved to be more reliable for particular insect groups (14), which indicates that regions within the cox gene have potential for accurate identification of *Phyllophaga* species and should be evaluated. The 5’ end region of the Cytochrome c oxidase subunit 1 (cox1) identified by the primers of Folmer et al. (24) has been extensively studied for distinguishing species, and there are also other regions with potential including one amplified by the primers ‘Pat’ and ‘Jerry’ (25), and one within Cytochrome b (CytB) amplified by primers ‘CB3’ and ‘CB4’ (22). In addition, a fragment of 28S nuclear rRNA containing the variable domains D3–D6 (identified using primers ‘FF’ and ‘DD’ [18, 26]) has potential for distinguishing coleopteran species. Therefore, combining morphology and DNA sequence analyses for adult identification, and then comparing DNA information from adults with larvae, would enable a more accurate taxonomic status of Phyllophaga species in maize crops.

Mexico is considered a center of Scarabaeidae diversity (27). Understanding the association between larvae and adults is important. However, this process requires maintaining the collected larvae to reach the adult stage to confirm taxonomic status (13), which can be time consuming as the larval stage can last more than 150 days in some species of *Phyllophaga* (28). DNA-based taxonomy may provide a valuable tool to make associations between different life stages (16, 18, 21). To determine the relationship between species diversity of *Phyllophaga* adults and larvae, in maize crops from different localities in the central region of Mexico. First, adults were identified using morphological characters and genetic markers to distinguish between species. Then, genetic information from adults was correlated with DNA of larvae, to infer spatial distribution and potential succession of species in larvae from the same places.

## Material and methods

### Larval collection and processing

**Figure 1:**
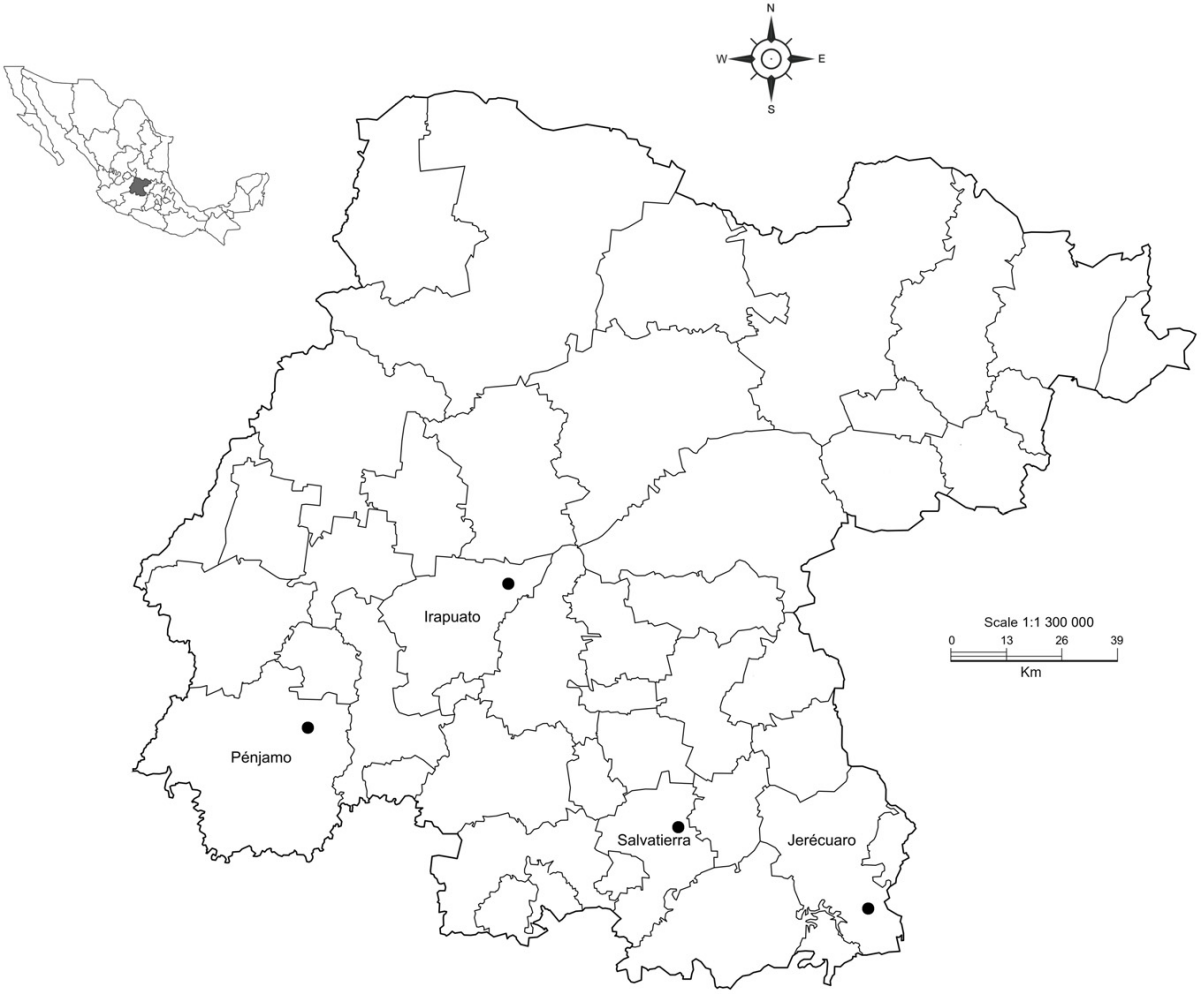
Map showing the four localities sampled in our study in Guanajuato state, Mexico.

White grub larvae were collected from different maize crops at five municipalities in Guanajuato state from September to December 2021 (**Table 1**, **Figure 1**). Approximately 50 larvae were collected on 1–3 dates per site. Larvae were collected following the methods described by Guzmán-Franco et al. (28). Briefly, third instar white grub larvae were collected manually from soil and deposited in a 70 × 40 × 20 cm plastic container filled with damp peat moss (Growing Mix^®^, Canada) and transported to the laboratory. Collected larvae were identified to genus based on the presence of palidia on the last abdominal segment (raster) and anal aperture morphology (27, 29). Only *Phyllophaga* larvae were selected for further analysis. Twenty larvae per location and sampling date were selected and immediately frozen at −20°C prior to further processing, but never longer than a month.

**Table 1:** Sampling sites and date where *Phyllophaga* adults and larvae were collected in the estate of Guanajuato, Mexico.

Municipality	Locality	Latitude	Longitude	Year	Month	Developmental stage collected
Penjamo	Tierras Negras	20.48110 N	−101.72766 W	2022	June	Adults (L1)
				2021	September	Larvae (3)
				2021	October	Larvae (6)
				2021	November	Larvae (9)
Irapuato	El Garbanzo	20.80464 N	−101.16668 W	2022	August	Adults (L6)
				2022	August	Adults (L7)
				2021	September	Larvae (2)
				2021	October	Larvae (5)
				2021	November	Larvae (10)
				2021	December	Larvae (8)
Jerecuaro	Puruagua	20.07275 N	−100.45883 W	2022	June	Adults (L4)
				2021	September	Larvae (4)
				2021	November	Larvae (7)
Salvatierra	El Caracol	20.19420 N	−100.78184 W	2022	June	Adults (L2)
				2022	June	Adults (L5)
				2022	August	Adults (L8)
				2021	September	Larvae (1)

All sampling sites were maize crops. The letter and numbers in brackets after the developmental stage represent the lot number, and this was used to identify each individual sample and sequence.

### Adult collection, processing and morphological identification

Adults were collected using light traps. For this, a 100 watts bulb was attached to a wooden post placed 1.5 m above the ground at each sampling location (**Table 1**, **Figure 1**). A 1.5 × 1.5 m transparent plastic screen fastened by two 1.5 m-long wooden stakes was placed behind each light bulb. In front of the plastic screen and beneath the light bulb, an 80×40×40 cm plastic container filled with soapy water was placed to collect the adults. To achieve at least 50 adults per light trap they were run from dusk until dawn over 24–36 h. Light traps were placed close to each of the sites where larvae had been collected but also close to a house to obtain an electricity supply. Adults were removed from the soapy water and deposited into 96% ethanol in 50 mL sterile centrifuge tubes and transported to the laboratory. Adults were collected between June and August 2022, one season after larval collection, assuming adults collected would belong to the same cohort as the larvae collected the previous year.

Adults were first identified to species level based on morphological attributes using taxonomic keys (1, 7, 30–34). From the 103 adults analysed, successful morphological identification was achieved for 45 specimens, the rest could not be accurately identified, either because critical morphological structures were damaged, or they were female.

### Molecular methods

#### DNA extraction

For adults, DNA was extracted from individual head capsules after they had been cut into small pieces. DNA was extracted using the Tissue & Insect DNA MicroPrep kit (Zymo Research, Irvine, CA, USA) following the manufacturer’s instructions. For DNA extraction from larvae, they were retrieved from −20°C, allowed to defrost at room temperature and rinsed with sterile distilled water to remove any attached peat moss. Only larval head capsules were used. Inside a sterile laminar flow chamber, the mandibles were removed using a sterile scalpel, and the cephalic capsule cut into small pieces. DNA was extracted using the same extraction kit as for adults. DNA concentration was estimated in each sample using a NanoDrop™ (Thermo Fisher Scientific, Inc. Waltham, MA, USA), and adjusted to 20 ng µL^−1^.

#### PCR and sequencing

Partial sequences of the mitochondrial genes cox1 and CytB were obtained. For the cox1 region, two sets of primers were used, LCO1490 (5’-GGT CAA CAA ATC ATA AAG ATA TTG G-3’), HCO2198 (5’-TAA ACT TCA GGG TGA CCA AAA AAT CA-3’) (24) and Pat (5’-TCC AAT GCA CTA ATC TGC CAT ATT A-3’), Jerry (5’-CAA CAT TTA TTT TGA TTT TTT GG-3’) (25). For CytB, the primers CB3 (5’-GAG GAG CAA CTG TAA TTA CTA A-3’) and CB4 (5’-AAA AGA AAR TAT CAT TCA GGT TGA AT-3’) (35) were used. A fragment of 28S nuclear rRNA containing the variable domains D3-D6 was amplified using the primers FF (5′-TTA CAC ACT CCT TAG CGG AT-3’) and DD (5′-GGG ACC CGT CCT TGA AAC AC-3’) (26). All these primers were used for adult DNA, whereas for larvae, only the primer pairs LCO1490-HCO2198 and CB3-CB4 were used based on the greater resolution obtained with adult DNA by these two primer pairs (see results). Reactions were made in a final volume of 30 µL containing 1X PCR buffer (Tris-Cl, KCl, (NH_4_)2SO_4_, 15 mmol L^–1^ MgCl_2_; pH 8.7), 0.2 µM of each primer, 0.2 mM of dNTPs, 0.5 U of *Taq*DNA polymerase (Qiagen^®^, GmbH, Hilden, Germany), 1 mM of MgCl_2_ and 3 μL (approx. 30 ng) of DNA. Amplifications were done using a T100™ Thermal Cycler (Bio-Rad Laboratories Inc., Hercules, CA, USA). Thermal conditions for the primer pair LCO1490-HCO2198 were one cycle of 60 s at 94°C, five cycles of 60 s at 94°C, 90 s at 45°C and 90 s at 72°C; 35 cycles of 60 s at 94°C, 90 s at 60°C and 60 s at 72°C with a final extension of 5 min at 72°C. For the primers CB3-CB4 conditions were one cycle of 5 min at 94°C; 35 cycles of 30 s at 95°C, 40 s at 50°C and 2 min at 72°C with a final extension of 10 min at 72°C. For the primer pairs Jerry-Pat and FF-DD conditions were one cycle of 3 min at 94°C; 35 cycles of 60 s at 94°C, 60 s at 48°C and 90 s at 72°C with a final extension of 5 min at 72°C. All PCR products were visualized on 1.5% agarose gels in 1×TAE buffer. Gels were stained with ethidium bromide (0.1 µg mL^−1^) and photographed. All PCR products were sent to Macrogen Inc. (South Korea) for direct sequencing.

#### Phylogenetic and genetic distances analyses

Sequences were edited manually using BioEdit (36). Multiple sequence alignments were made using the Clustal W software (37) implemented in BioEdit. Model tests and inference cladograms were done in IQ TREE using maximum likelihood analysis (38). The robustness of branches was estimated by bootstrap approximation analysis with 5000 repeated samples using UFBoot2 (39). An independent analysis was conducted for the sequence data generated by each molecular marker, and in each case, sequences of *Diplotaxis* sp. (Coleoptera: Melolonthidae) were used as an outgroup. Phylogenetic trees were edited and visualized using iTOL (40). Analyses of the pairwise genetic distances between and within nucleotide sequences of the species studied were done in MEGA 11 (41) using the Kimura two-parameter (K2P) model (42) for each molecular marker, and for adult and larvae separately. Standard error estimates were obtained by a bootstrap procedure with 1000 replicates. A threshold of 2% genetic divergence was considered among species, for cox1 sequences (43) and CytB (44). All sequences were deposited in GenBank; see **Supplementary Table S1** for accession numbers.

Additional species delimitation analyses using two methods were performed. Bayesian implementation of the Poisson tree processes (bPTP) model (45) was performed using the webserver available at http://species.h-its.org/ptp, with default parameters. This analysis is based on the postulate that the number of substitutions among species is greater than that within species. For the second method, two analyses were done, ‘automatic barcode gap discovery’ analysis (ABGD) (46) using the webserver available at https://bioinfo.mnhn.fr/abi/public/abgd/abgdweb.html, and ‘assemble species by automatic partitioning’ analysis (ASAP) (47) using the webserver available at https://bioinfo.mnhn.fr/abi/public/asap/asapweb.html, both using the Kimura´s two parameter as the substitution model (42), to propose partitions of species based on pairwise genetic distances among DNA sequences. These two analyses assume that the amount of genetic variation within species is smaller than among species (48).

Adult identifications were made by combining the phylogenetic analysis results with morphological identification. Sequences from adult specimens, for which their identity had been confirmed by both molecular and morphological analysis, were included in the set of larval sequences to facilitate determining species within the genus *Phyllophaga*. With the adult and larval species information, a qualitative description of the distribution of adult species in the sampled sites was produced; and using the larval information, changes in species composition over time were described at the sampled sites.

## Results

### Species determination of *Phyllophaga* adults and distribution

**Figure 2:**
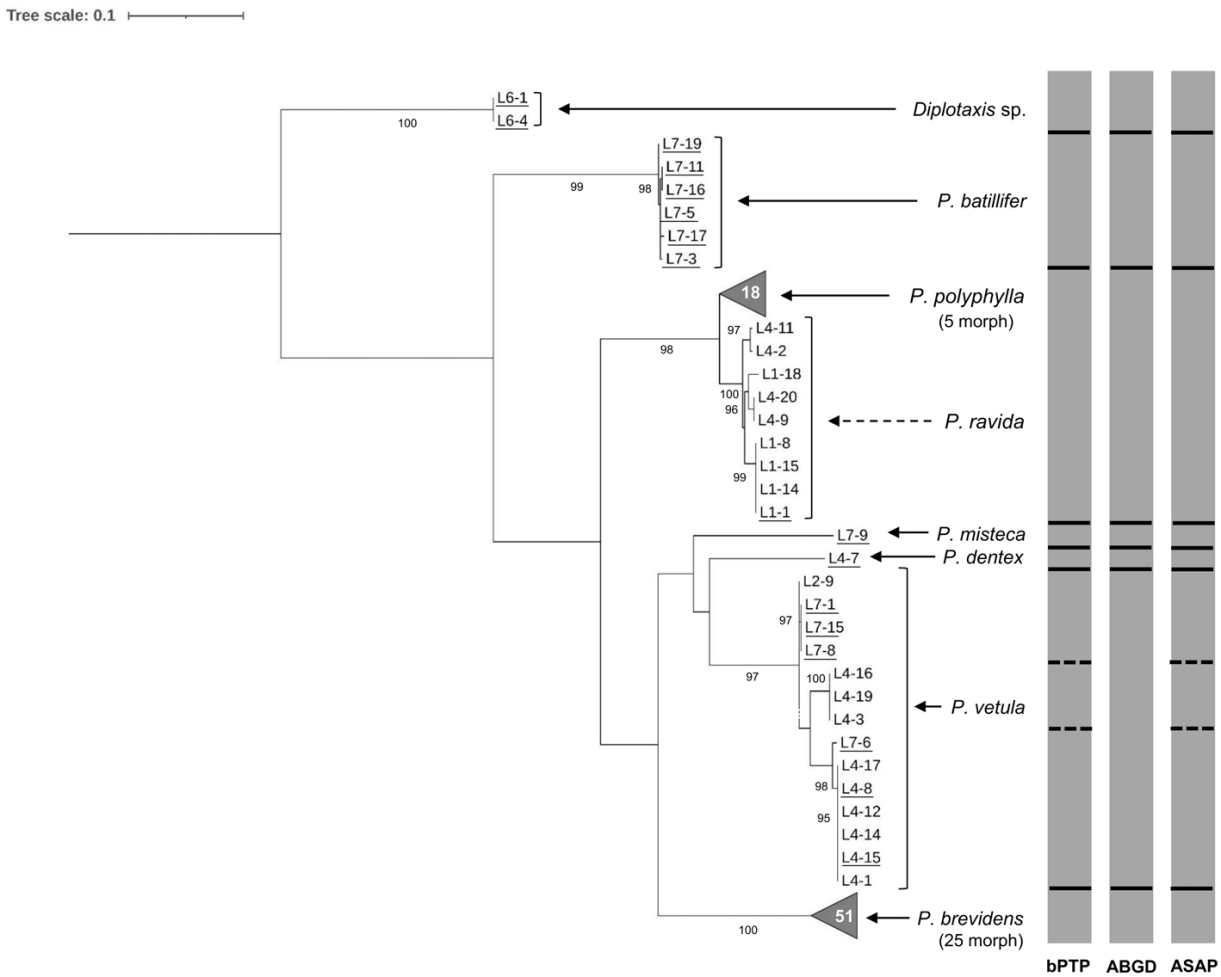
Phylogeny of species from the genus *Phyllophaga* obtained in this study, inferred from maximum-likelihood analysis of partial sequences of the cox1 gene acquired from adult specimens using HCO-LCO primers. Underlined samples were also identified morphologically. Gray bars represent results of bPTP, ABGD and ASAP analyses. Solid lines within gray bars represent a consistent result with all three analyses. Dashed line were used when species differentiation was not obtained with all three analyses. The number inside the gray triangles represents the number of sequences contained in that branch which has been collapsed for better visual presentation. GenBank accession numbers for all specimens are shown in **Supplementary Table S1**. Only bootstrap values > 85% are shown. The scale bar corresponds to 0.1 nucleotide substitutions per site.

Morphological identification of adults showed the presence of seven *Phyllophaga* species: *P. polyphylla* (Bates), *P. brevidens* (Bates), *P. vetula* (Horn), *P. batillifer* (Bates), *P. misteca* (Bates), *P. dentex* (Bates) and *P. ravida* (Blanchard) (Coleoptera: Melolonthidae). Overall, analysis of the 100 sequences obtained with HCO-LCO primers confirmed the presence of the same seven morphologically identified *Phyllophaga* species. Bootstrap analysis showed separation between all species with values above 90% (**Figure 2**). However, analyses by bPTP, ABGD and ASAP failed to distinguish between *P. ravida* and *P. polyphylla* (**Figure 2**); genetic distance analyses showed a 2.56% separation between these two species (**Table 2**) which was the smallest genetic divergence amongst *Phyllophaga* species but still confirmed the genetic separation between *P. ravida* and *P. polyphylla* (**Table 2**). A high genetic divergence was found amongst *P. vetula* sequences, as shown by bootstrap values above 90% distinguishing three groups within this species (**Figure 2**). Also, three analyses (bPTP, ABGD and ASAP) showed the presence of three potential cryptic species within *P. vetula* (**Figure 2**). The intraspecific genetic distance amongst *P. vetula* was 2.09% (**Table 2**). Overall, interspecific genetic distances amongst the other *Phyllophaga* species were between 11 and 21% (**Table 2**). The within-species genetic distances estimated for all species, except *P. vetula*, ranged from 0.14 to 1.00%, confirming that all sequences allocated in each group were from the same species.

**Table 2:** Percentage of K2P genetic distances with standard error (SE) for sequences from adults.

HCO-LCO sequences (cox1)									
Group	1*. P. misteca*	2. *P. polyphylla*	3. *P. ravida*	4. *P. vetula*	5. *P. brevidens*	6. *P. dentex*	7. *P. batillifer*	8. *Diplotaxis*	WSGD
1	N/C								N/C
2	15.55 (1.98)								0.36 (0.13)
3	16.30 (2.01)	2.52 (0.67)							1.00 (0.31)
4	11.49 (1.61)	14.25 (1.85)	14.51 (1.85)						2.09 (0.49)
5	17.28 (2.15)	14.05 (1.88)	15.06 (1.94)	13.60 (1.80)					0.68 (0.25)
6	13.20 (1.82)	13.60 (1.82)	12.94 (1.76)	11.31 (1.60)	14.15 (1.94)				N/C
7	21.06 (2.38)	17.89 (2.12)	19.03 (2.20)	20.77 (2.27)	17.40 (2.12)	19.72 (2.30)			0.14 (0.10)
8	22.72 (2.51)	23.45 (2.53)	24.10 (2.58)	21.25 (2.36)	22.92 (2.43)	22.41 (2.52)	22.14 (2.45)	N/C	00.00 (0.00)

Pat-Jerry sequences (cox1)									
Group	1*. P. vetula*	2. *P. batillifer*	3. *P. polyphylla*	4. *P. misteca*	5. *P. brevidens*	6. *P. ravida*	7. *P. dentex*	8. *Diplotaxis*	WSGD
1	N/C								1.81 (0.35)
2	19.93 (1.88)								0.06 (0.06)
3	17.39 (1.75)	18.84 (1.97)							0.38 (0.14)
4	13.74 (1.57)	22.78 (2.18)	17.22 (1.87)						N/C
5	14.69 (1.61)	19.12 (1.93)	15.07 (1.63)	15.29 (1.71)					0.45 (0.18)
6	17.99 (1.79)	19.26 (1.95)	1.86 (0.74)	16.95 (1.86)	14.88 (1.61)				0.91 (0.24)
7	14.45 (1.63)	19.65 (1.97)	17.48 (1.88)	14.59 (1.70)	16.19 (1.73)	16.96 (1.80)			N/C
8	22.78 (2.08)	22.55 (2.22)	19.99 (2.03)	24.47 (2.29)	20.99 (2.07)	20.30 (1.99)	22.88 (2.24)	N/C	0.16 (0.16)

CB3-CB4 sequences (CytB)									
Group	1*. P. ravida*	2. *P. brevidens*	3. *P. vetula*	4. *P. polyphylla*	5. *P. dentex*	6. *P. batillifer*	7. *Diplotaxis*	WSGD
1	N/C							1.89 (0.52)
2	20.80 (2.77)							1.01 (0.36)
3	22.10 (2.88)	16.62 (2.28)						2.43 (0.58)
4	4.63 (1.04)	20.41 (2.75)	21.76 (2.79)					1.46 (0.48)
5	23.15 (3.08)	13.66 (2.14)	14.38 (2.19)	21.56 (2.91)				N/C
6	24.32 (3.12)	20.27 (2.78)	18.78 (2.58)	23.77 (3.04)	18.16 (2.65)			0.15 (0.15)
7	30.16 (3.56)	31.39 (3.60)	28.60 (3.33)	30.82 (3.62)	29.36 (3.45)	26.44 (3.15)	N/C	N/C

FF-DD sequences (28S)									
Group	1*. P. ravida*	2. *P. polyphylla*	3. *P. brevidens*	4. *P. vetula*	5. *P. batillifer*	6. *P. misteca*	7. *Diplotaxis*	WSGD
1	N/C							0.00 (0.00)
2	0.00 (0.00)							0.00 (0.00)
3	0.23 (0.23)	0.23 (0.23)						0.01 (0.01)
4	0.00 (0.00)	0.00 (0.00)	0.23 (0.23)					0.00 (0.00)
5	0.47 (0.33)	0.47 (0.33)	0.70 (0.40)	0.47 (0.33)				0.00 (0.00)
6	0.00 (0.00)	0.00 (0.00)	0.23 (0.23)	0.00 (0.00)	0.47 (0.33)			N/C
7	2.87 (0.85)	2.87 (0.85)	2.63 (0.82)	2.87 (0.85)	2.87 (0.85)	2.87 (0.85)	N/C	N/C

WSGD, within species genetic distances. N/C, not calculated.

**Figure 3:**
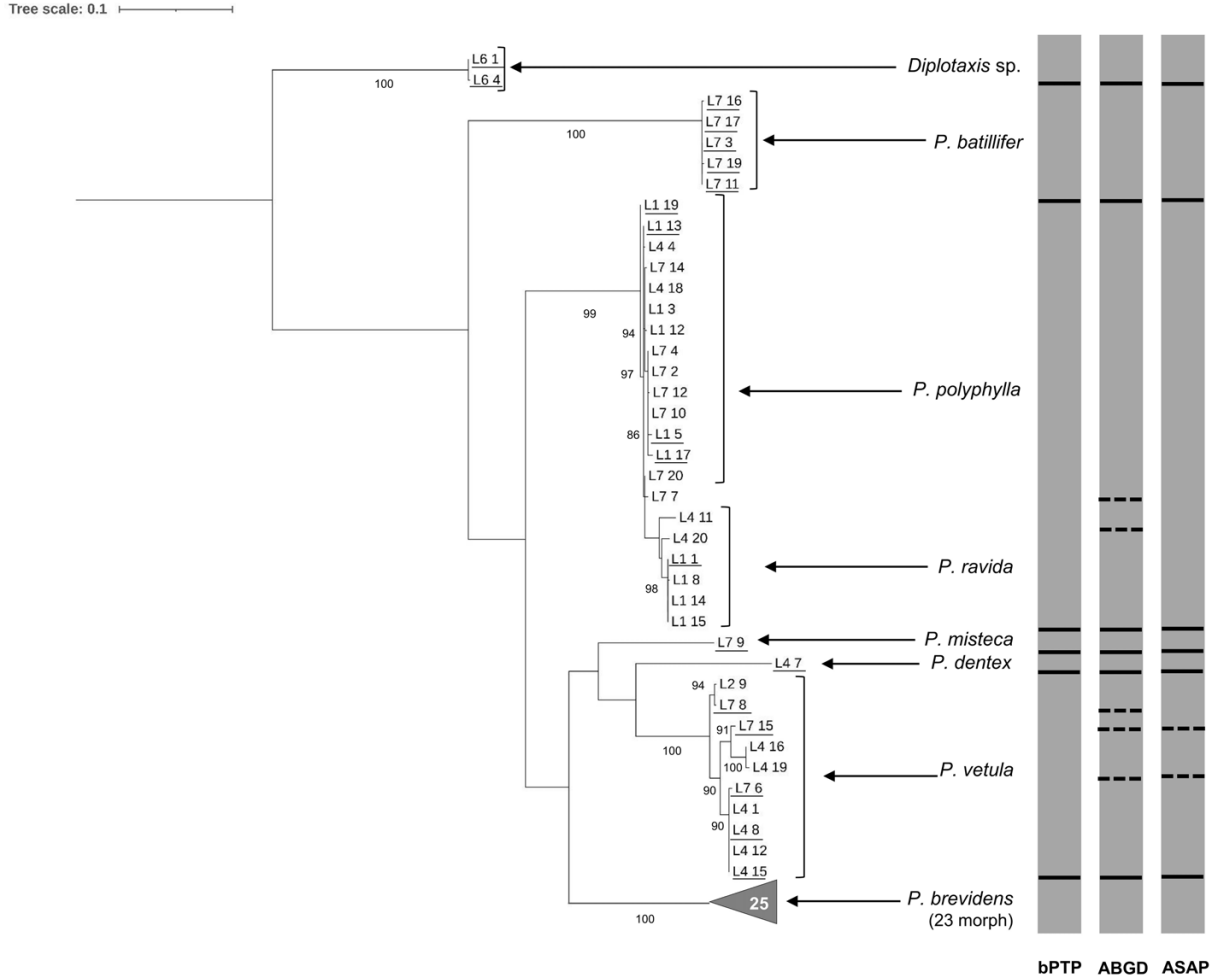
Phylogeny of species from the genus *Phyllophaga* obtained in this study, inferred from maximum-likelihood analysis of partial sequences of the cox1 gene acquired from adult specimens using Pat-Jerry primers. Underlined samples were also identified morphologically. Gray bars represent results of bPTP, ABGD and ASAP analyses. Solid lines within gray bars represent a consistent result with all three analyses. Dashed lines were used when species differentiation was not obtained with all three analyses. The number inside the gray triangles represents the number of sequences contained in that branch which has collapsed for better visual presentation. GenBank accession numbers for all specimens are shown in **Supplementary Table S1**. Only bootstrap values > 85% are shown. The scale bar corresponds to 0.1 nucleotide substitutions per site.

Similar results were found when the 63 sequences obtained using Pat-Jerry primers were analysed with bootstrap values above 90% (**Figure 3**). ABGD analysis separated *P. ravida* sequences from *P. polyphylla*. ABGD and ASAP analyses confirmed the potential cryptic species in *P vetula* (**Figure 3**). Interspecific distance analysis showed a range between 13.74% to 22.78% separation amongst species, but only 1.86% separation between *P. polyphylla* and *P. ravida* (**Table 2**). When intra specific distances were estimated, all were below 1% except for *P. vetula* which was 1.81% (**Table 2**).

**Figure 4:**
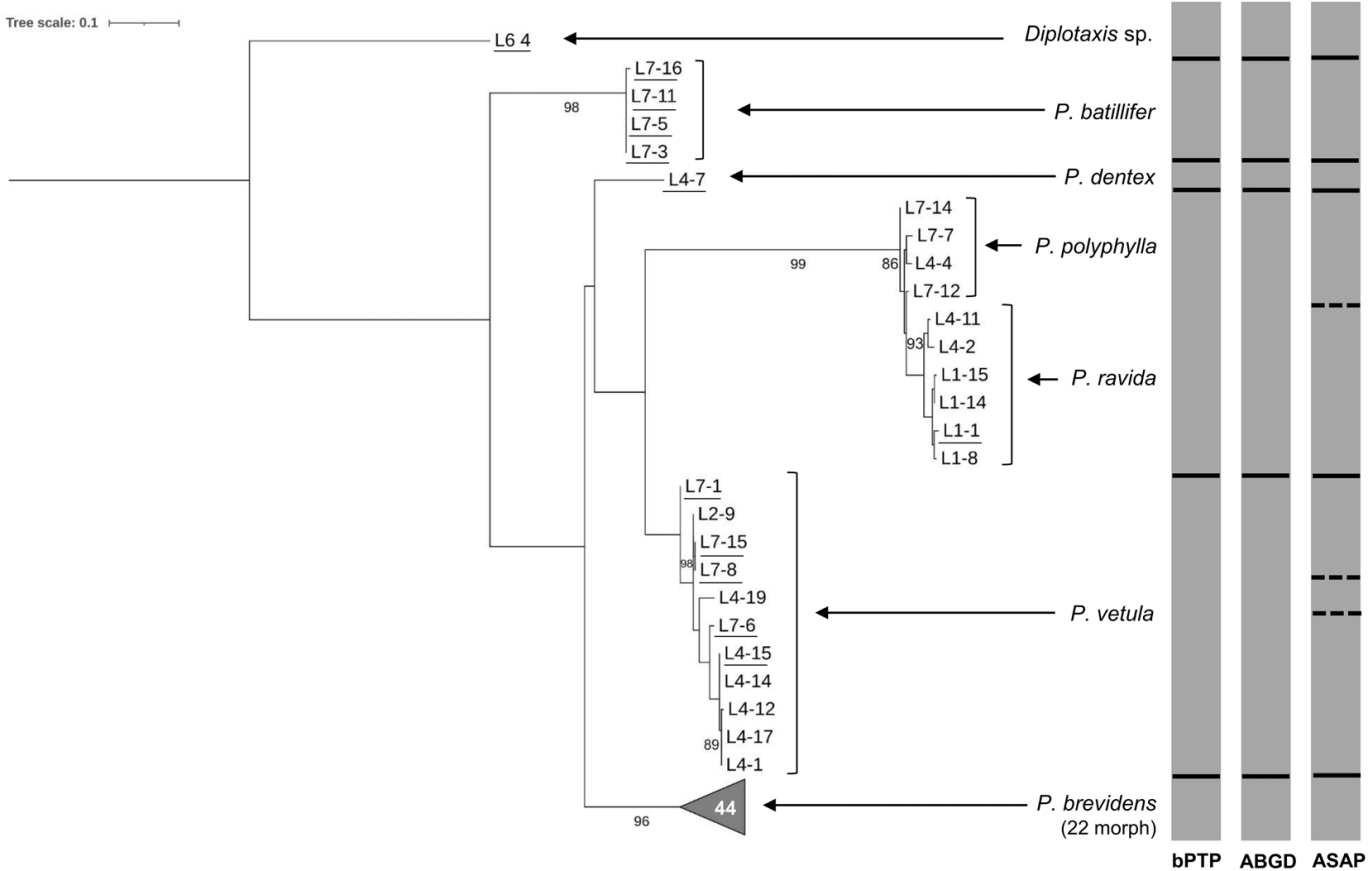
Phylogeny of species from the genus *Phyllophaga* obtained in this study, inferred from maximum-likelihood analysis of partial sequences of the CytB gene acquired from adult specimens using CB3-CB4 primers. Underlined samples were also identified morphologically. Gray bars represent results of bPTP, ABGD and ASAP analyses. Solid lines within gray bars represent a consistent result with all three analyses. Dashed lines were used when species differentiation was not obtained with all three analyses. The number inside the gray triangles represents the number of sequences contained in that branch which has collapsed for better visual presentation. GenBank accession numbers for all specimens are shown in **Supplementary Table S1**. Only bootstrap values > 85% are shown. The scale bar corresponds to 0.1 nucleotide substitutions per site.

The 70 sequences obtained using CB3-CB primers on the CytB gene distinguished between the seven species identified morphologically with bootstrap values above 90% (**Figure 4**). Here, ASAP analysis showed a clear separation between *P. polyphylla* and *P. ravida*, as well as the potential cryptic species within *P. vetula* (**Figure 4**). Interspecific distance analyses showed a separation amongst species of 4.63% to 24.32%. Genetic divergence between *P. polyphylla* and *P. ravida* was 4.63%, providing a larger and clearer separation between these two species than with the previous primer set sequences (**Table 2**). Within species separation was always below 2% except for *P. vetula* which was 2.43% (**Table 2**). The results obtained with the 28S primers for the 58 sequences did not achieve successful separation amongst *Phyllophaga* species; all interspecific genetic distances were below 0.5%, with a distance above 2% only found between *Phyllophaga* species and *Diplotaxis* (the outgroup) (**Table 2**).

**Figure 5:**
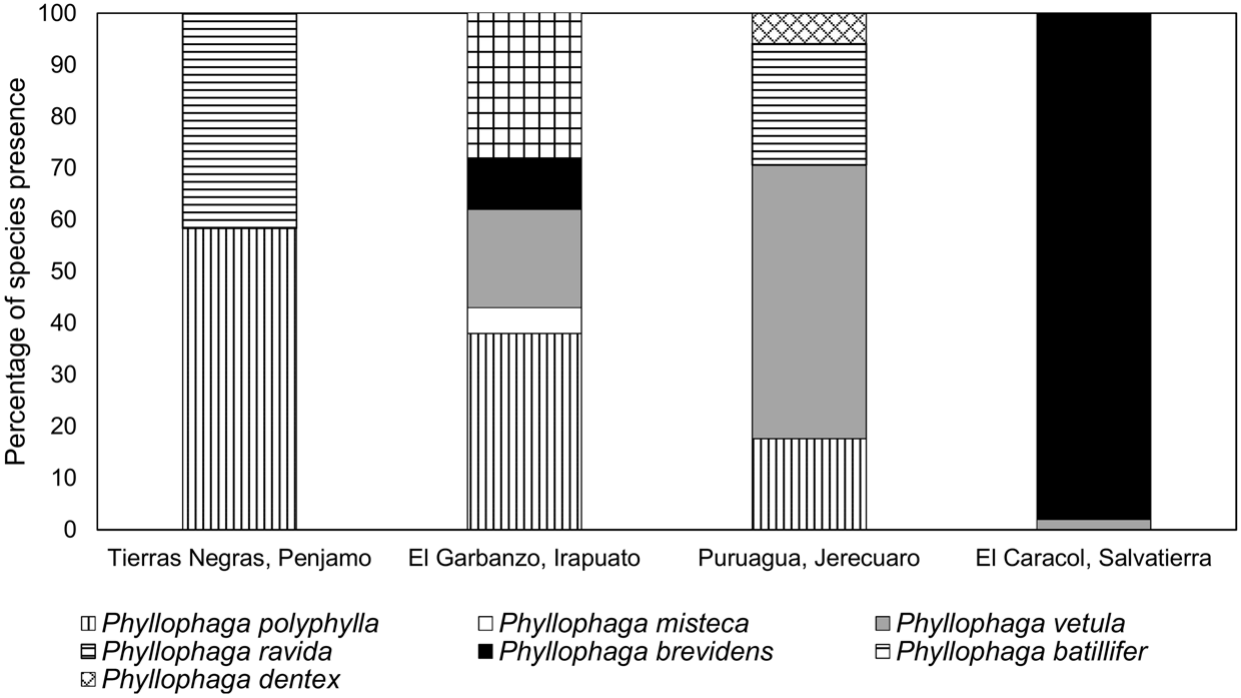
Presence (%) of different *Phyllophaga* species found at each locality sampled based on adult data. More information about the sampling sites can be found in **Table 1**.

Based on adult sequences, *P. polyphylla*, was the most widely distributed species as it was found at three of four sampled locations (**Figure 5**). El Caracol, was the only site sampled with almost entirely one species, *P. brevidens*. El Garbanzo was the site with the greatest number of species, *P. batillifer, P. polyphylla, P. vetula, P. brevidens* and *P. misteca* (**Figure 5**).

### Distribution and species dynamics of *Phyllophaga* larvae

**Figure 6:**
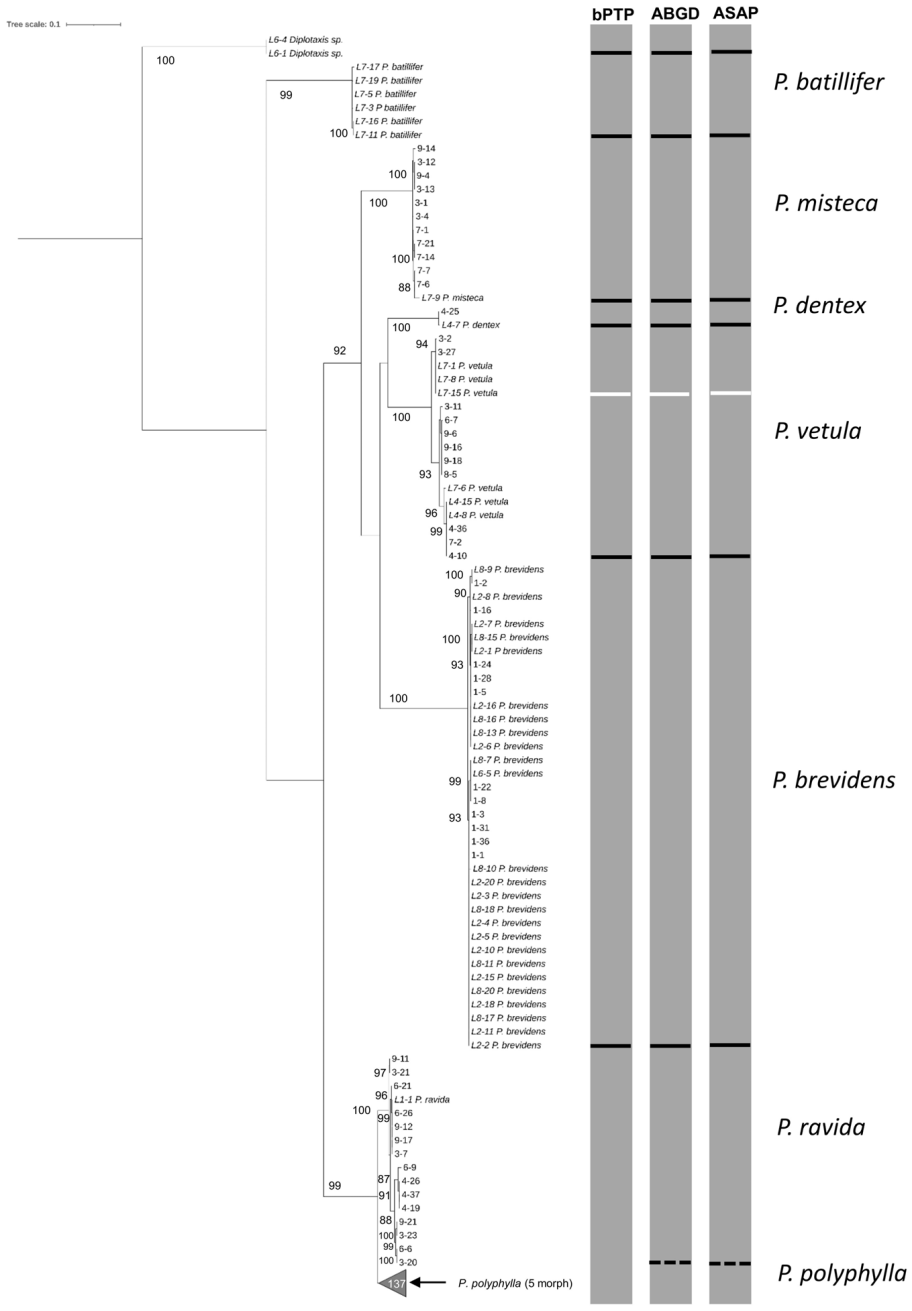
Phylogeny of species from the genus *Phyllophaga* obtained in this study, inferred from maximum-likelihood analysis of partial sequences of the cox1 gene acquired from larvae using HCO-LCO primers. Samples with the taxonomic species are sequences from adults used as reference. Gray bars represent results of bPTP, ABGD and ASAP analyses. Solid black lines within gray bars represent consistent results with all three analyses. Solid white lines within gray bars suggest cryptic species within *P. vetula*. Dashed lines were used when species differentiation was not obtained with all three analyses. The number inside the gray triangles represents the number of sequences contained in that branch which was collapsed for better visual presentation. GenBank accession numbers for all specimens are shown in **Supplementary Table S1**. Only bootstrap values > 85% are shown. The scale bar corresponds to 0.1 nucleotide substitutions per site.

The analyses done on 180 sequences larvae plus 47 sequences from adults obtained with the HCO-LCO primers, showed the presence of six *Phyllophaga* species, *P. polyphylla*, *P. brevidens*, *P. vetula*, *P. misteca*, *P. dentex* and *P. ravida* with bootstrap values above 94% (**Figure 6**); this was confirmed by the three analyses, bPTP, ABGD and ASAP, which showed the potential presence of cryptic species within *P. vetula* (**Figure 6**). No *P. batillifer* larvae were found at any sampling site. Genetic distance analyses showed a divergence between 2.39% to 20.62% amongst species, with the smallest separation between *P. polyphylla* and *P. ravida* (2.39%); all intraspecific divergences were below 2% (**Table 3**).

**Table 3:** Percentage of K2P genetic distances with standard error (SE) for sequences from larvae and adults.

HCO-LCO sequences (cox1)									
Group	1. *P. batillifer*	2*. P. brevidens*	3*. P. polyphylla*	4. *P. ravida*	5. *P. misteca*	6*. P. dentex*	7. *P. vetula*	8. *Diplotaxis*	WSGD
1	N/C								0.12 (0.08)
2	16.34 (1.89)								1.29 (0.25)
3	17.29 (1.93)	13.08 (1.68)							0.27 (0.08)
4	18.03 (2.00)	14.01 (1.71)	2.39 (0.60)						0.90 (0.27)
5	19.78 (2.13)	15.76 (1.84)	14.67 (1.85)	15.87 (1.94)					0.47 (0.17)
6	18.37 (2.07)	12.96 (1.61)	13.18 (1.65)	12.37 (1.59)	12.19 (1.62)				N/C
7	20.62 (2.19)	13.65 (1.68)	13.91 (1.73)	14.07 (1.72)	11.65 (1.55)	11.07 (1.55)			1.80 (0.38)
8	21.28 (2.21)	22.56 (2.34)	22.82 (2.42)	23.13 (2.41)	21.83 (2.41)	21.78 (2.39)	20.73 (2.91)	N/C	N/C
CB3-CB4 sequences (CytB)									
Group	1. *P. batillifer*	2*. P. brevidens*	3*. P. polyphylla*	4. *P. dentex*	5. *P. ravida*	6*. P. misteca*	7. *P. vetula*	8. *Diplotaxis*	WSGD
1	N/C								0.00 (0.00)
2	21.23 (3.85)								0.85 (0.47)
3	26.19 (4.24)	21.86 (3.92)							1.12 (0.46)
4	18.10 (3.58)	15.08 (3.16)	23.23 (4.07)						N/C
5	26.63 (4.46)	20.81 (3.79)	3.67 (1.30)	25.19 (4.27)					0.51 (0.29)
6	19.93 (3.62)	15.52 (3.24)	18.76 (3.40)	15.01 (3.14)	19.87 (3.66)				0.83 (0.42)
7	18.93 (3.43)	16.02 (3.18)	23.19 (3.88)	16.03 (3.13)	24.56 (4.17)	15.83 (3.08)			2.25 (0.77)
8	23.43 (3.79)	29.94 (4.75)	29.75 (4.91)	28.07 (4.57)	30.36 (5.12)	27.91 (4.56)	27.49 (4.36)	N/C	N/C

Sequences from adults were used as a reference. WSGD, within species genetic distances. N/C, not calculated.

**Figure 7:**
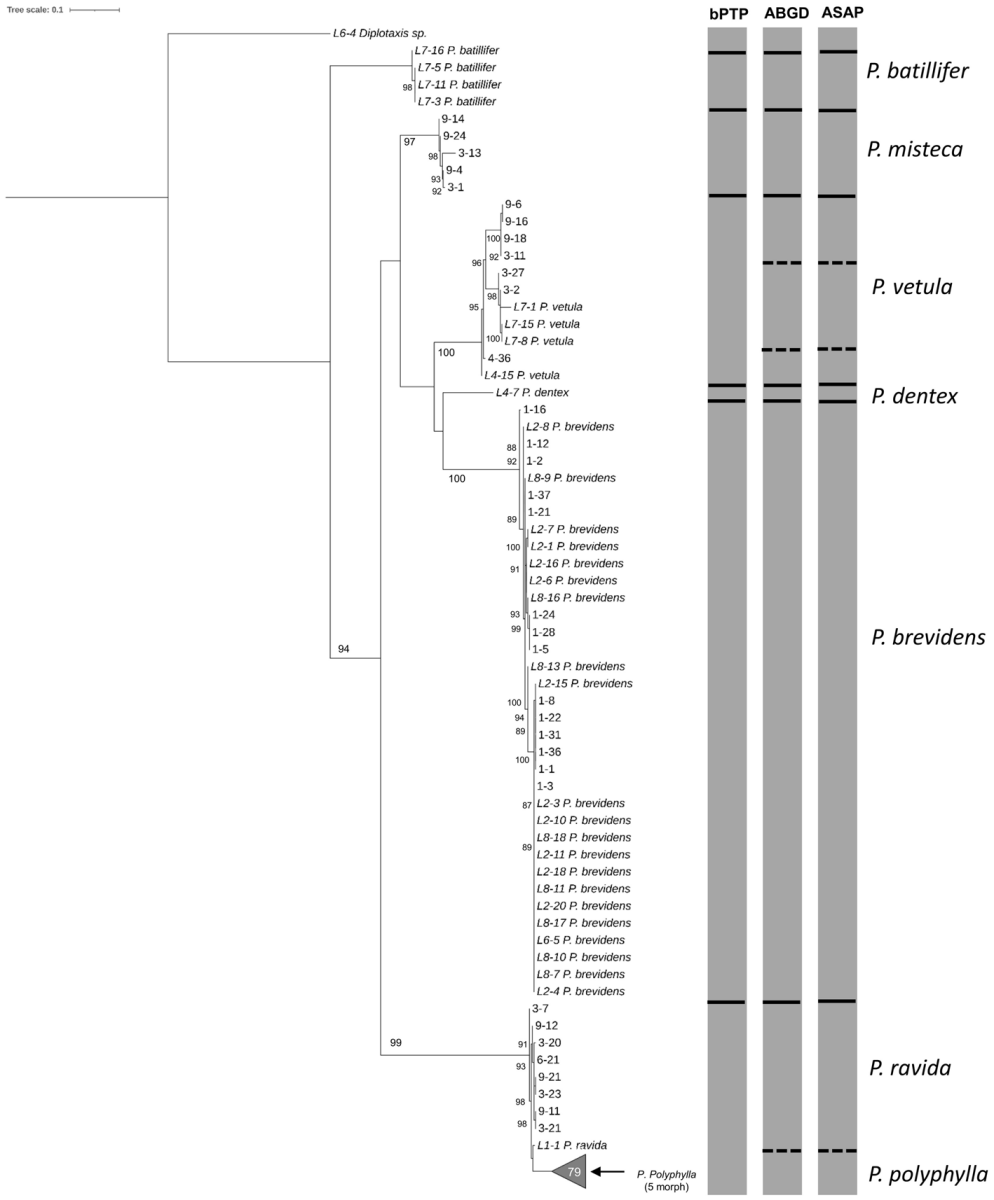
Phylogeny of species from the genus *Phyllophaga* obtained in this study, inferred from maximum-likelihood analysis of partial sequences of the CytB gene acquired from larvae using CB3-CB4 primers. Samples with the taxonomic species are sequences from adults used as reference. Gray bars represent results of bPTP, ABGD and ASAP analyses. Solid lines within gray bars represent a consistent result with all three analyses. Dashed lines were used when species differentiation was not obtained with all three analyses. The number inside the gray triangles represents the number of sequences contained in that branch which was collapsed for better visual presentation. GenBank accession numbers for all specimens are shown in **Supplementary Table S1**. Only bootstrap values > 85% are shown. The scale bar corresponds to 0.1 nucleotide substitutions per site.

Analyses of 109 sequences larvae plus 36 sequences from adults of the CytB region using CB3-CB4 primers showed similar results with all six species being identified with bootstrap values above 90% (**Figure 7**). Only bPTP analysis did not separate between *P. polyphylla* and *P. ravida*, while ABGD and ASAP confirmed the separation between these close species, and the presence of cryptic species in *P. vetula* (**Figure 7**). Overall, genetic distances amongst *Phyllophaga* species ranged between 3.67% and 23.19%, with the smallest separation being 3.67% between *P. ravida* and *P. polyphylla* (**Table 3**). Intraspecific distances for all species were always below 2%, except for *P. vetula* which had a divergence of 2.25% (**Table 3**).

**Figure 8:**
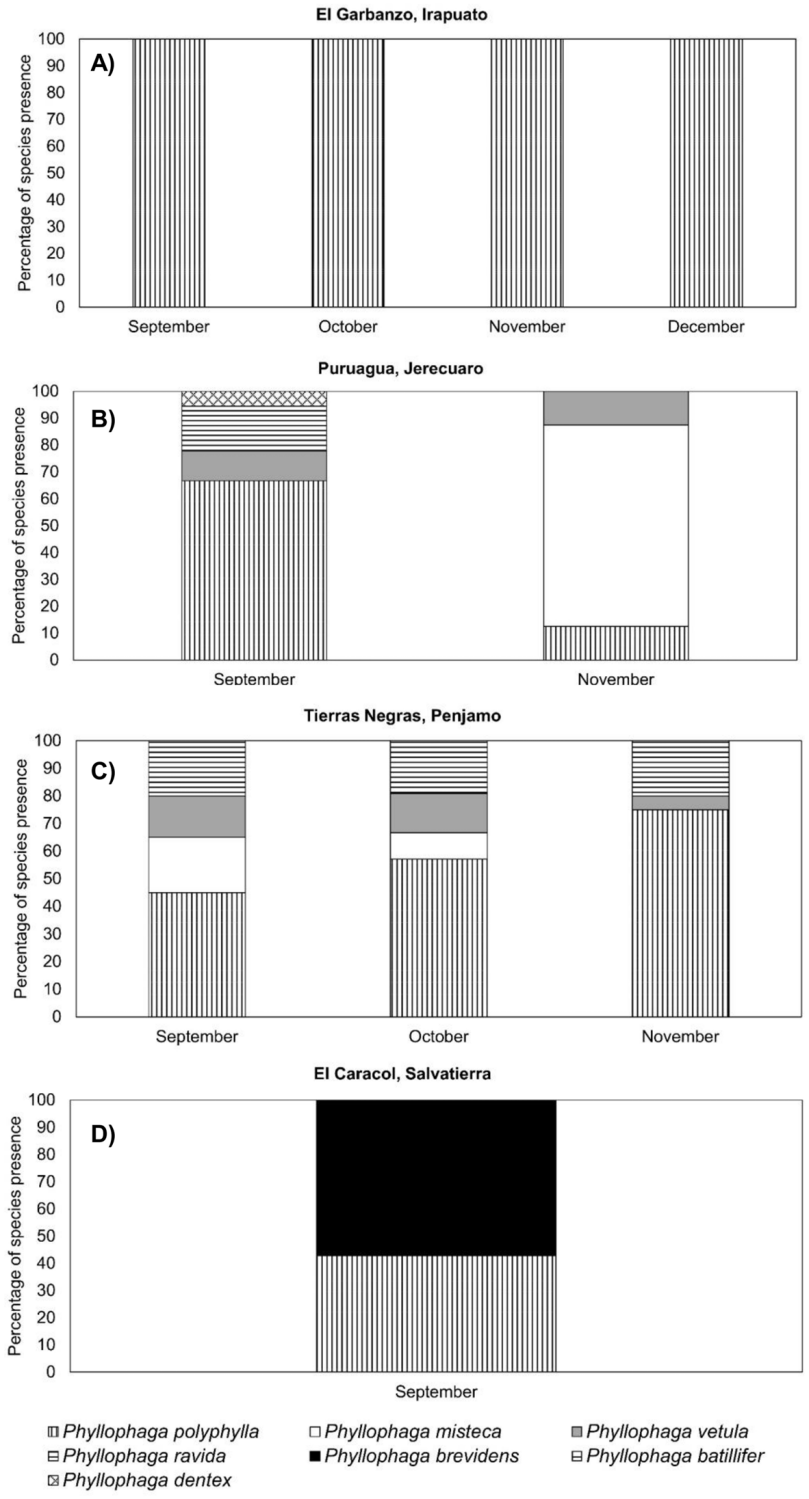
Presence (%) of different species of *Phyllophaga* larvae at different sampling times. **(A)** El Garbanzo, **(B)** Puruagua, **(C)** Tierras Negras and **(D)** El Caracol. More information about the sampling sites can be found in **Table 1**.

At the El Garbanzo sampling site, we only found *P. polyphylla* on the four sampling dates (**Figure 8A**). At the Puruagua site, in the September sampling, the predominant species was *P. polyphylla* representing 65% of those collected, followed by *P. ravida, P. vetula* and *P. dentex* with approx. 10% each (**Figure 8B**). In November, the predominant species was *P. misteca*, which was not detected in September; *P. misteca* accounted for 80% of those collected, followed by *P. polyphylla* and *P. vetula* with 10% each (**Figure 8B**). At the Tierras Negras sampling site, the predominant species on all three sampling dates was *P. polyphylla* representing 45%, 55% and 75% of those collected in September, October and November, respectively; the other two species found were *P. ravida*, which maintained a 20% presence on all sampling dates, followed by *P. vetula* with 15% presence down to 5% in November, and *P. misteca* which was present in September representing 20% of the population, reducing to 5% in October and disappearing in November (**Figure 8C**). At El Caracol, 55% of the population was *P. brevidens* and 45% was *P. polyphylla* (**Figure 8D**).

## Discussion

Our results showed that molecular taxonomy could be an accurate and reliable method to distinguish between species of *Phyllophaga* in maize crops, as recently reported for other *Phyllophaga* species in lawns (49). In adults, this approach helped to identify damaged specimens and females, both of which cannot easily be identified morphologically. Then, using adult sequences for comparison, the species of larvae were also successfully determined. For adults, sequences obtained using the HCO-LCO, Pat-Jerry (*cox*) and the CB3-CB4 (CytB) primers, distinguished between species according to the morphological identification. It is clear from our results that *P. ravida* and *P. poyphylla* are very closely related species with just 2.56% separation between them in the HCO-LCO region, but with greater separation in the CB3-CB4 region of the CytB gene (4.63%) (**Table 2**). Results were similar for sequences from larvae (combined with adult sequences), with 2.39% and 3.67% separation for the HCO-LCO and CB3-CB4 regions, respectively (**Table 3**). These results are not surprising as the CytB gene has been previously used to separate close species and detect genetic variation within the same species more efficiently compared with cox1, as reported recently for sandfly species (Diptera: Psychodidae) (50). Both regions provide consistent results for the other *Phyllophaga* species. Sequences from the D3-D6 domain of the 28S nuclear rRNA gene did not successfully distinguish between *Phyllophaga* species, a result that was expected as this gene has a low evolution rate compared with mitochondrial genes, and it is used more commonly to determine higher taxa rather than species (51, 52). CytB sequences have been used successfully to determine species (33). The fact that CytB sequences provided more accurate discrimination between *P. ravida* and *P. polyphylla* could be because the CytB gene has a greater percentage of variable positions compared with the cox1 region (53).

From our results, it is also very clear that there are at least three cryptic species could be found within *P. vetula* (**Figures 2**–**4**, **6**, **7**). For example, for the LCO-HCO sequences (**Figure 2**), the first group contained samples from El Caracol and El Garbanzo (L2 and L7 respectively), the second group with samples only from Puruagua, (starting with L4), and the last group from “El Garbanzo” and Puruagua (**Supplementary Table S1**), suggesting that geographical origin does not play an important role.

It is interesting that more species were found in adult samples than in larval samples. For example, at El Garbanzo, larval taxonomy showed that only *P. polyphylla* was present in all months sampled (**Figure 8A**) while when adults from the same sampling site were analysed five species were found (**Figure 5**). Adults were captured using light traps, so it is possible that the other species were attracted from other crops outside the maize crop studied, overestimating the number of species found on maize (19). However, we also observed at other sampling sites that the number of species found in larvae was greater than in adults. For example, at Tierras Negras, the most abundant species were *P. polyphylla* and *P. ravida* on all sampling dates but *P. vetula* and *P. misteca* were present at the early sampling dates and gradually disappeared leaving only *P. polyphylla* and *P. ravida* at the end (**Figure 8C**); perhaps only these two species were observed as adults as a result of this (**Figure 5**). At El Caracol larvae were collected only on one date in September, when two species were present, *P. polyphylla* and *P. brevidens* (**Figure 8D**); of the adults collected practically all specimens were *P. brevidens* (**Figure 5**), despite sampling in two months (**Table 1**). It is clear from our results that the species composition of *Phyllophaga* in all sites sampled changes over time, as shown in the results for larvae. The exact cause of this succession is unclear, but it may be related to the phenological stage of the plants, changes in the chemical composition of soils and more specifically in the rhizosphere (e.g. 54) which may favour the persistence of some species over others. Based on our results, we suggest that species diversity studies should be based on larval taxonomy achieved using sequencing in combination with adult morphological and genetic taxonomy. For adults, we believe using other methods such as emergence traps (e.g. 55) for sampling would better reflect the species diversity at the location.

In conclusion, molecular taxonomy provided a powerful tool for accurate species determination in the genus *Phyllophaga*, especially for larvae, in combination with morphological identification for more accurate species determination and diversity assessment. The most abundant and widely distributed species in the central region of Mexico and in the sampled sites was *P. polyphylla*, followed by *P. brevidens*, as the most abundant although it was only found at one site (El Caracol). The existence of cryptic species within *P. vetula* needs to be investigated further. Larval molecular taxonomy enabled us to observe species turnover. Determining species diversity in larvae, which are the most damaging stage to plants, will allow further studies on potential differential susceptibility of the different species found to different control strategies, which could lead to more successful population reduction of these pests in maize.

## Data Availability

The datasets presented in this study can be found in online repositories. The names of the repository/repositories and accession number(s) can be found in the article/**Supplementary Material**.
