# Supplementary material for: A DNA-based approach to infer species diversity of larvae and adults from the white grub genus Phyllophaga (Coleoptera: Scarabeidae)
Source: Front Insect Sci. 2024 Nov 7;4:1465794. doi: 10.3389/finsc.2024.1465794 (PMC11579710; doi:10.3389/finsc.2024.1465794)
Supplement: Supplementary file 1 [file Table1.docx]

**Supplementary tables**

Table 1S. GenBank accession numbers for partial sequences obtained from adults and larvae of *Phyllophaga* used for analysis. All samples were collected in maize crops. Larvae were collected in 2021 and adults in 2022.

| **Adults** | | | | | | | | |
| --- | --- | --- | --- | --- | --- | --- | --- | --- |
| **Specimen** | **Species** | **HCO-LCO** | **Pat-Jerry** | | **CB3-CB4** | **28S** | **Locality** | **Municipality** |
| L7-3 | *Phyllophaga batillifer* | PQ231208 | PQ219982 | | PQ210210 | PQ226398 | El Garbanzo | Irapuato |
| L7-5 | *P. batillifer* | PQ231209 |  | | PQ210211 |  | El Garbanzo | Irapuato |
| L7-11 | *P. batillifer* | PQ231210 | PQ219983 | | PQ210212 | PQ226399 | El Garbanzo | Irapuato |
| L7-16 | *P. batillifer* | PQ231211 | PQ219984 | | PQ210213 | PQ226400 | El Garbanzo | Irapuato |
| L7-17 | *P. batillifer* | PQ231212 | PQ219985 | |  | PQ226401 | El Garbanzo | Irapuato |
| L7-19 | *P. batillifer* | PQ231213 | PQ219986 | |  | PQ226402 | El Garbanzo | Irapuato |
| L2-1 | *Phyllophaga brevidens* | PQ231214 | PQ219987 | | PQ210214 | PQ226403 | El Caracol | Salvatierra |
| L2-2 | *P. brevidens* | PQ231215 | PQ219988 | |  | PQ226404 | El Caracol | Salvatierra |
| L2-3 | *P. brevidens* | PQ231216 | PQ219989 | | PQ210215 | PQ226405 | El Caracol | Salvatierra |
| L2-4 | *P. brevidens* | PQ231217 | PQ219990 | | PQ210216 |  | El Caracol | Salvatierra |
| L2-5 | *P. brevidens* | PQ231218 | PQ219991 | |  | PQ226406 | El Caracol | Salvatierra |
| L2-6 | *P. brevidens* | PQ231219 | PQ219992 | | PQ210217 | PQ226407 | El Caracol | Salvatierra |
| L2-7 | *P. brevidens* | PQ231220 | PQ219993 | | PQ210218 | PQ226408 | El Caracol | Salvatierra |
| L2-8 | *P. brevidens* | PQ231221 | PQ219994 | | PQ210219 | PQ226409 | El Caracol | Salvatierra |
| L2-9 | *P. brevidens* |  |  | |  | PQ226410 | El Caracol | Salvatierra |
| L2-10 | *P. brevidens* | PQ231222 | PQ219995 | | PQ210220 | PQ226411 | El Caracol | Salvatierra |
| L2-11 | *P. brevidens* | PQ231223 | PQ219996 | | PQ210221 | PQ226412 | El Caracol | Salvatierra |
| L2-12 | *P. brevidens* |  |  | |  | PQ226413 | El Caracol | Salvatierra |
| L2-13 | *P. brevidens* |  |  | |  | PQ226414 | El Caracol | Salvatierra |
| L2-14 | *P. brevidens* | PQ231224 |  | | PQ210222 | PQ226415 | El Caracol | Salvatierra |
| L2-15 | *P. brevidens* | PQ231225 | PQ219997 | | PQ210223 | PQ226416 | El Caracol | Salvatierra |
| L2-16 | *P. brevidens* | PQ231226 | PQ219998 | | PQ210224 | PQ226417 | El Caracol | Salvatierra |
| L2-17 | *P. brevidens* | PQ231227 |  | | PQ210225 | PQ226418 | El Caracol | Salvatierra |
| L2-18 | *P. brevidens* | PQ231228 | PQ219999 | | PQ210226 | PQ226419 | El Caracol | Salvatierra |
| L2-19 | *P. brevidens* |  |  | |  | PQ226420 | El Caracol | Salvatierra |
| L2-20 | *P. brevidens* | PQ231229 | PQ220000 | | PQ210227 | PQ226421 | El Caracol | Salvatierra |
| L5-1 | *P. brevidens* | PQ231230 |  | |  |  | El Caracol | Salvatierra |
| L5-7 | *P. brevidens* | PQ231231 |  | | PQ210228 |  | El Caracol | Salvatierra |
| L5-8 | *P. brevidens* | PQ231232 |  | | PQ210229 | PQ226422 | El Caracol | Salvatierra |
| L5-9 | *P. brevidens* | PQ231233 | PQ220001 | | PQ210230 |  | El Caracol | Salvatierra |
| L5-10 | *P. brevidens* | PQ231234 |  | |  |  | El Caracol | Salvatierra |
| L5-11 | *P. brevidens* | PQ231235 |  | | PQ210231 | PQ226423 | El Caracol | Salvatierra |
| L5-12 | *P. brevidens* | PQ231236 |  | | PQ210232 |  | El Caracol | Salvatierra |
| L5-13 | *P. brevidens* | PQ231237 |  | | PQ210233 |  | El Caracol | Salvatierra |
| L5-14 | *P. brevidens* | PQ231238 |  | | PQ210234 | PQ226424 | El Caracol | Salvatierra |
| L5-15 | *P. brevidens* | PQ231239 |  | | PQ210235 | PQ226425 | El Caracol | Salvatierra |
| L5-16 | *P. brevidens* | PQ231240 |  | | PQ210236 | PQ226426 | El Caracol | Salvatierra |
| L5-17 | *P. brevidens* | PQ231241 |  | | PQ210237 | PQ226427 | El Caracol | Salvatierra |
| L5-18 | *P. brevidens* | PQ231242 |  | | PQ210238 |  | El Caracol | Salvatierra |
| L5-19 | *P. brevidens* | PQ231243 |  | | PQ210239 | PQ226428 | El Caracol | Salvatierra |
| L5-20 | *P. brevidens* | PQ231244 |  | | PQ210240 |  | El Caracol | Salvatierra |
| L6-2 | *P. brevidens* | PQ231245 | PQ220002 | | PQ210241 | PQ226429 | El Garbanzo | Irapuato |
| L6-3 | *P. brevidens* |  | PQ220003 | |  |  | El Garbanzo | Irapuato |
| L6-5 | *P. brevidens* | PQ231246 | PQ220004 | | PQ210242 |  | El Garbanzo | Irapuato |
| L8-1 | *P. brevidens* | PQ231247 |  | | PQ210243 | PQ226430 | El Caracol | Salvatierra |
| L8-3 | *P. brevidens* | PQ231248 |  | | PQ210245 |  | El Caracol | Salvatierra |
| L8-4 | *P. brevidens* | PQ231249 |  | |  |  | El Caracol | Salvatierra |
| L8-5 | *P. brevidens* | PQ231250 |  | | PQ210246 | PQ226431 | El Caracol | Salvatierra |
| L8-6 | *P. brevidens* | PQ231251 |  | |  |  | El Caracol | Salvatierra |
| L8-7 | *P. brevidens* | PQ231252 | PQ220005 | | PQ210247 | PQ226432 | El Caracol | Salvatierra |
| L8-8 | *P. brevidens* | PQ231253 |  | | PQ210248 | PQ226433 | El Caracol | Salvatierra |
| L8-9 | *P. brevidens* | PQ231254 |  | | PQ210249 | PQ226434 | El Caracol | Salvatierra |
| L8-10 | *P. brevidens* | PQ231255 | PQ220006 | | PQ210250 |  | El Caracol | Salvatierra |
| L8-11 | *P. brevidens* | PQ231256 | PQ220007 | | PQ210251 |  | El Caracol | Salvatierra |
| L8-12 | *P. brevidens* | PQ231257 |  | |  |  | El Caracol | Salvatierra |
| L8-13 | *P. brevidens* | PQ231258 |  | | PQ210252 | PQ226435 | El Caracol | Salvatierra |
| L8-14 | *P. brevidens* | PQ231259 |  | | PQ210253 |  | El Caracol | Salvatierra |
| L8-15 | *P. brevidens* | PQ231260 | PQ220008 | |  |  | El Caracol | Salvatierra |
| L8-16 | *P. brevidens* | PQ231261 | PQ220009 | | PQ210254 | PQ226436 | El Caracol | Salvatierra |
| L8-17 | *P. brevidens* | PQ231262 | PQ220010 | | PQ210255 |  | El Caracol | Salvatierra |
| L8-18 | *P. brevidens* | PQ231263 | PQ220011 | | PQ210256 |  | El Caracol | Salvatierra |
| L8-19 | *P. brevidens* |  |  | | PQ210257 | PQ226437 | El Caracol | Salvatierra |
| L8-20 | *P. brevidens* | PQ231264 |  | |  |  | El Caracol | Salvatierra |
| L4-7 | *Phyllophaga dentex* | PQ231265 | PQ220012 | | PQ210258 |  | Puruagua | Jerecuaro |
| L7-9 | *Phyllophaga misteca* | PQ231266 | PQ220013 | |  | PQ226438 | El Garbanzo | Irapuato |
| L1-3 | *Phyllophaga polyphylla* | PQ231267 | PQ220014 | |  |  | Tierras Negras | Penjamo |
| L1-5 | *P. polyphylla* | PQ231268 | PQ220015 | |  |  | Tierras Negras | Penjamo |
| L1-12 | *P. polyphylla* | PQ231269 | PQ220016 | |  | PQ226439 | Tierras Negras | Penjamo |
| L1-13 | *P. polyphylla* | PQ231270 | PQ220017 | |  | PQ226440 | Tierras Negras | Penjamo |
| L1-16 | *P. polyphylla* | PQ231271 |  | |  | PQ226441 | Tierras Negras | Penjamo |
| L1-17 | *P. polyphylla* | PQ231272 | PQ220018 | |  |  | Tierras Negras | Penjamo |
| L1-19 | *P. polyphylla* | PQ231273 | PQ220019 | |  |  | Tierras Negras | Penjamo |
| L4-4 | *P. polyphylla* | PQ231274 | PQ220020 | | PQ210259 |  | Puruagua | Jerecuaro |
| L4-13 | *P. polyphylla* | PQ231275 |  | |  |  | Puruagua | Jerecuaro |
| L4-18 | *P. polyphylla* | PQ231276 | PQ220021 | |  | PQ226442 | Puruagua | Jerecuaro |
| L7-2 | *P. polyphylla* | PQ231277 | PQ220022 | |  | PQ226443 | El Garbanzo | Irapuato |
| L7-4 | *P. polyphylla* | PQ231278 | PQ220023 | |  | PQ226444 | El Garbanzo | Irapuato |
| L7-7 | *P. polyphylla* | PQ231279 | PQ220024 | | PQ210260 |  | El Garbanzo | Irapuato |
| L7-10 | *P. polyphylla* | PQ231280 | PQ220025 | |  | PQ226445 | El Garbanzo | Irapuato |
| L7-12 | *P. polyphylla* | PQ231281 | PQ220026 | | PQ210261 |  | El Garbanzo | Irapuato |
| L7-14 | *P. polyphylla* | PQ231282 | PQ220027 | | PQ210262 |  | El Garbanzo | Irapuato |
| L7-18 | *P. polyphylla* | PQ231283 |  | |  | PQ226446 | El Garbanzo | Irapuato |
| L7-20 | *P. polyphylla* | PQ231284 | PQ220028 | |  |  | El Garbanzo | Irapuato |
| L1-1 | *Phyllophaga ravida* | PQ231285 | PQ220029 | | PQ210263 | PQ226447 | Tierras Negras | Penjamo |
| L1-8 | *P. ravida* | PQ231286 | PQ220030 | | PQ210264 | PQ226448 | Tierras Negras | Penjamo |
| L1-14 | *P. ravida* | PQ231287 | PQ220031 | | PQ210265 | PQ226449 | Tierras Negras | Penjamo |
| L1-15 | *P. ravida* | PQ231288 | PQ220032 | | PQ210266 | PQ226450 | Tierras Negras | Penjamo |
| L1-18 | *P. ravida* | PQ231289 |  | |  | PQ226451 | Tierras Negras | Penjamo |
| L4-2 | *P. ravida* | PQ231290 |  | | PQ210267 |  | Puruagua | Jerecuaro |
| L4-9 | *P. ravida* | PQ231291 |  | |  |  | Puruagua | Jerecuaro |
| L4-11 | *P. ravida* | PQ231292 | PQ220033 | | PQ210268 |  | Puruagua | Jerecuaro |
| L4-20 | *P. ravida* | PQ231293 | PQ220034 | |  |  | Puruagua | Jerecuaro |
| L2-9 | *Phyllophaga vetula* | PQ231294 | PQ220035 | | PQ210269 |  | El Caracol | Salvatierra |
| L4-1 | *P. vetula* | PQ231295 | PQ220036 | | PQ210270 |  | Puruagua | Jerecuaro |
| L4-3 | *P. vetula* | PQ231296 |  | |  |  | Puruagua | Jerecuaro |
| L4-8 | *P. vetula* | PQ231297 | PQ220037 | |  |  | Puruagua | Jerecuaro |
| L4-12 | *P. vetula* | PQ231298 | PQ220038 | | PQ210271 |  | Puruagua | Jerecuaro |
| L4-14 | *P. vetula* | PQ231299 |  | | PQ210272 |  | Puruagua | Jerecuaro |
| L4-15 | *P. vetula* | PQ231300 | PQ220039 | | PQ210273 |  | Puruagua | Jerecuaro |
| L4-16 | *P. vetula* | PQ231301 | PQ220040 | |  | PQ226452 | Puruagua | Jerecuaro |
| L4-17 | *P. vetula* | PQ231302 |  | | PQ210274 |  | Puruagua | Jerecuaro |
| L4-19 | *P. vetula* | PQ231303 | PQ220041 | | PQ210275 |  | Puruagua | Jerecuaro |
| L7-1 | *P. vetula* | PQ231304 |  | | PQ210276 | PQ226453 | El Garbanzo | Irapuato |
| L7-6 | *P. vetula* | PQ231305 | PQ220042 | | PQ210277 | PQ226454 | El Garbanzo | Irapuato |
| L7-8 | *P. vetula* | PQ231306 | PQ220043 | | PQ210278 | PQ226455 | El Garbanzo | Irapuato |
| L7-15 | *P. vetula* | PQ231307 | PQ220044 | | PQ210279 |  | El Garbanzo | Irapuato |
| L6-1 | *Diplotaxis sp.* | PQ231308 | PQ220045 | |  |  | El Garbanzo | Irapuato |
| L6-4 | *Diplotaxis sp.* | PQ231309 | PQ220046 | | PQ210280 | PQ226456 | El Garbanzo | Irapuato |
| **Larvae** | | | | | | | | |
| **Specimen** | **Species** | **HCO-LCO** | | **CB3-CB4** | | **Locality** | **Municipality** | **Month** |
| 1-1 | *Phyllophaga brevidens* | PQ221277 | | PQ246916 | | El caracol | Salvatierra | Septiembre |
| 1-2 | *P. brevidens* | PQ221278 | | PQ246917 | | El caracol | Salvatierra | Septiembre |
| 1-3 | *P. brevidens* | PQ221279 | | PQ246918 | | El caracol | Salvatierra | Septiembre |
| 1-5 | *P. brevidens* | PQ221280 | | PQ246919 | | El caracol | Salvatierra | Septiembre |
| 1-6 | *P. brevidens* | PQ221281 | |  | | El caracol | Salvatierra | Septiembre |
| 1-8 | *P. brevidens* | PQ221282 | | PQ246920 | | El caracol | Salvatierra | Septiembre |
| 1-12 | *P. brevidens* |  | | PQ246921 | | El caracol | Salvatierra | Septiembre |
| 1-16 | *P. brevidens* | PQ221283 | | PQ246922 | | El caracol | Salvatierra | Septiembre |
| 1-21 | *P. brevidens* |  | | PQ246923 | | El caracol | Salvatierra | Septiembre |
| 1-22 | *P. brevidens* | PQ221284 | | PQ246924 | | El caracol | Salvatierra | Septiembre |
| 1-24 | *P. brevidens* | PQ221285 | | PQ246925 | | El caracol | Salvatierra | Septiembre |
| 1-28 | *P. brevidens* | PQ221286 | | PQ246926 | | El caracol | Salvatierra | Septiembre |
| 1-31 | *P. brevidens* | PQ221287 | | PQ246927 | | El caracol | Salvatierra | Septiembre |
| 1-34 | *P. brevidens* |  | |  | | El caracol | Salvatierra | Septiembre |
| 1-36 | *P. brevidens* | PQ221288 | | PQ246928 | | El caracol | Salvatierra | Septiembre |
| 1-37 | *P. brevidens* |  | | PQ246929 | | El caracol | Salvatierra | Septiembre |
| 4-25 | *Phyllophaga dentex* | PQ221289 | |  | | Puruagua | Jerecuaro | Septiembre |
| 3-1 | *Phyllophaga misteca* | PQ221290 | | PQ246930 | | Tierras Negras | Penjamo | Septiembre |
| 3-4 | *P. misteca* | PQ221291 | |  | | Tierras Negras | Penjamo | Septiembre |
| 3-12 | *P. misteca* | PQ221292 | |  | | Tierras Negras | Penjamo | Septiembre |
| 3-13 | *P. misteca* | PQ221293 | | PQ246931 | | Tierras Negras | Penjamo | Septiembre |
| 7-1 | *P. misteca* | PQ221294 | |  | | Puruagua | Jerecuaro | Noviembre |
| 7-6 | *P. misteca* | PQ221295 | |  | | Puruagua | Jerecuaro | Noviembre |
| 7-7 | *P. misteca* | PQ221296 | |  | | Puruagua | Jerecuaro | Noviembre |
| 7-10 | *P. misteca* | PQ221297 | |  | | Puruagua | Jerecuaro | Noviembre |
| 7-14 | *P. misteca* | PQ221298 | |  | | Puruagua | Jerecuaro | Noviembre |
| 7-21 | *P. misteca* | PQ221299 | |  | | Puruagua | Jerecuaro | Noviembre |
| 9-4 | *P. misteca* | PQ221300 | | PQ246932 | | Tierras Negras | Penjamo | Octubre |
| 9-14 | *P. misteca* | PQ221301 | | PQ246933 | | Tierras Negras | Penjamo | Octubre |
| 9-24 | *P. misteca* |  | | PQ246934 | | Tierras Negras | Penjamo | Octubre |
| 1-9 | *P. polyphylla* | PQ221302 | | PQ246935 | | El caracol | Salvatierra | Septiembre |
| 1-12 | *P. polyphylla* |  | |  | | El caracol | Salvatierra | Septiembre |
| 1-13 | *P. polyphylla* | PQ221303 | | PQ246936 | | El caracol | Salvatierra | Septiembre |
| 1-21 | *P. polyphylla* |  | |  | | El caracol | Salvatierra | Septiembre |
| 1-26 | *P. polyphylla* | PQ221304 | | PQ246937 | | El caracol | Salvatierra | Septiembre |
| 1-29 | *P. polyphylla* | PQ221305 | |  | | El caracol | Salvatierra | Septiembre |
| 1-33 | *P. polyphylla* | PQ221306 | |  | | El caracol | Salvatierra | Septiembre |
| 1-34 | *P. polyphylla* | PQ221307 | | PQ246938 | | El caracol | Salvatierra | Septiembre |
| 2-1 | *P. polyphylla* | PQ221308 | |  | | El Garbanzo | Irapuato | Septiembre |
| 2-2 | *P. polyphylla* | PQ221309 | |  | | El Garbanzo | Irapuato | Septiembre |
| 2-4 | *P. polyphylla* | PQ221310 | | PQ246939 | | El Garbanzo | Irapuato | Septiembre |
| 2-9 | *P. polyphylla* | PQ221311 | | PQ246940 | | El Garbanzo | Irapuato | Septiembre |
| 2-10 | *P. polyphylla* | PQ221312 | | PQ246941 | | El Garbanzo | Irapuato | Septiembre |
| 2-11 | *P. polyphylla* | PQ221313 | | PQ246942 | | El Garbanzo | Irapuato | Septiembre |
| 2-21 | *P. polyphylla* | PQ221314 | | PQ246943 | | El Garbanzo | Irapuato | Septiembre |
| 2-22 | *P. polyphylla* | PQ221315 | |  | | El Garbanzo | Irapuato | Septiembre |
| 2-23 | *P. polyphylla* | PQ221316 | |  | | El Garbanzo | Irapuato | Septiembre |
| 2-25 | *P. polyphylla* | PQ221317 | | PQ246944 | | El Garbanzo | Irapuato | Septiembre |
| 2-28 | *P. polyphylla* | PQ221318 | |  | | El Garbanzo | Irapuato | Septiembre |
| 2-30 | *P. polyphylla* | PQ221319 | |  | | El Garbanzo | Irapuato | Septiembre |
| 2-32 | *P. polyphylla* | PQ221320 | | PQ246945 | | El Garbanzo | Irapuato | Septiembre |
| 2-33 | *P. polyphylla* | PQ221321 | | PQ246946 | | El Garbanzo | Irapuato | Septiembre |
| 2-34 | *P. polyphylla* | PQ221322 | | PQ246947 | | El Garbanzo | Irapuato | Septiembre |
| 2-36 | *P. polyphylla* | PQ221323 | |  | | El Garbanzo | Irapuato | Septiembre |
| 2-37 | *P. polyphylla* | PQ221324 | |  | | El Garbanzo | Irapuato | Septiembre |
| 2-38 | *P. polyphylla* | PQ221325 | |  | | El Garbanzo | Irapuato | Septiembre |
| 2-39 | *P. polyphylla* | PQ221326 | |  | | El Garbanzo | Irapuato | Septiembre |
| 2-41 | *P. polyphylla* | PQ221327 | |  | | El Garbanzo | Irapuato | Septiembre |
| 2-42 | *P. polyphylla* | PQ221328 | |  | | El Garbanzo | Irapuato | Septiembre |
| 3-3 | *P. polyphylla* | PQ221329 | | PQ246948 | | Tierras Negras | Penjamo | Septiembre |
| 3-9 | *P. polyphylla* | PQ221330 | | PQ246949 | | Tierras Negras | Penjamo | Septiembre |
| 3-14 | *P. polyphylla* | PQ221331 | |  | | Tierras Negras | Penjamo | Septiembre |
| 3-15 | *P. polyphylla* | PQ221332 | | PQ246950 | | Tierras Negras | Penjamo | Septiembre |
| 3-16 | *P. polyphylla* | PQ221333 | | PQ246951 | | Tierras Negras | Penjamo | Septiembre |
| 3-19 | *P. polyphylla* | PQ221334 | | PQ246952 | | Tierras Negras | Penjamo | Septiembre |
| 3-24 | *P. polyphylla* | PQ221335 | |  | | Tierras Negras | Penjamo | Septiembre |
| 3-25 | *P. polyphylla* | PQ221336 | |  | | Tierras Negras | Penjamo | Septiembre |
| 3-26 | *P. polyphylla* | PQ221337 | | PQ246953 | | Tierras Negras | Penjamo | Septiembre |
| 4-4 | *P. polyphylla* | PQ221338 | | PQ246954 | | Puruagua | Jerecuaro | Septiembre |
| 4-5 | *P. polyphylla* | PQ221339 | | PQ246955 | | Puruagua | Jerecuaro | Septiembre |
| 4-9 | *P. polyphylla* | PQ221340 | | PQ246956 | | Puruagua | Jerecuaro | Septiembre |
| 4-10 | *P. polyphylla* |  | | PQ246957 | | Puruagua | Jerecuaro |  |
| 4-21 | *P. polyphylla* | PQ221341 | |  | | Puruagua | Jerecuaro | Septiembre |
| 4-27 | *P. polyphylla* | PQ221342 | |  | | Puruagua | Jerecuaro | Septiembre |
| 4-28 | *P. polyphylla* | PQ221343 | |  | | Puruagua | Jerecuaro | Septiembre |
| 4-30 | *P. polyphylla* | PQ221344 | | PQ246958 | | Puruagua | Jerecuaro | Septiembre |
| 4-31 | *P. polyphylla* | PQ221345 | | PQ246959 | | Puruagua | Jerecuaro | Septiembre |
| 4-32 | *P. polyphylla* | PQ221346 | | PQ246960 | | Puruagua | Jerecuaro | Septiembre |
| 4-35 | *P. polyphylla* | PQ221347 | | PQ246961 | | Puruagua | Jerecuaro | Septiembre |
| 4-38 | *P. polyphylla* | PQ221348 | |  | | Puruagua | Jerecuaro | Septiembre |
| 4-42 | *P. polyphylla* | PQ221349 | | PQ246962 | | Puruagua | Jerecuaro | Septiembre |
| 5-3 | *P. polyphylla* | PQ221350 | | PQ246963 | | El Garbanzo | Irapuato | Octubre |
| 5-4 | *P. polyphylla* | PQ221351 | | PQ246964 | | El Garbanzo | Irapuato | Octubre |
| 5-5 | *P. polyphylla* | PQ221352 | | PQ246965 | | El Garbanzo | Irapuato | Octubre |
| 5-7 | *P. polyphylla* | PQ221353 | |  | | El Garbanzo | Irapuato | Octubre |
| 5-9 | *P. polyphylla* | PQ221354 | |  | | El Garbanzo | Irapuato | Octubre |
| 5-10 | *P. polyphylla* | PQ221355 | |  | | El Garbanzo | Irapuato | Octubre |
| 5-12 | *P. polyphylla* | PQ221356 | |  | | El Garbanzo | Irapuato | Octubre |
| 5-17 | *P. polyphylla* | PQ221357 | |  | | El Garbanzo | Irapuato | Octubre |
| 5-19 | *P. polyphylla* | PQ221358 | |  | | El Garbanzo | Irapuato | Octubre |
| 5-22 | *P. polyphylla* | PQ221359 | |  | | El Garbanzo | Irapuato | Octubre |
| 5-23 | *P. polyphylla* | PQ221360 | |  | | El Garbanzo | Irapuato | Octubre |
| 5-24 | *P. polyphylla* | PQ221361 | | PQ246966 | | El Garbanzo | Irapuato | Octubre |
| 5-25 | *P. polyphylla* | PQ221362 | | PQ246967 | | El Garbanzo | Irapuato | Octubre |
| 5-27 | *P. polyphylla* | PQ221363 | | PQ246968 | | El Garbanzo | Irapuato | Octubre |
| 5-28 | *P. polyphylla* | PQ221364 | | PQ246969 | | El Garbanzo | Irapuato | Octubre |
| 5-30 | *P. polyphylla* | PQ221365 | |  | | El Garbanzo | Irapuato | Octubre |
| 5-39 | *P. polyphylla* | PQ221366 | |  | | El Garbanzo | Irapuato | Octubre |
| 5-41 | *P. polyphylla* | PQ221367 | | PQ246970 | | El Garbanzo | Irapuato | Octubre |
| 6-2 | *P. polyphylla* |  | | PQ246971 | | Tierras Negras | Penjamo | Noviembre |
| 6-4 | *P. polyphylla* | PQ221368 | |  | | Tierras Negras | Penjamo | Noviembre |
| 6-5 | *P. polyphylla* | PQ221369 | | PQ246972 | | Tierras Negras | Penjamo | Noviembre |
| 6-10 | *P. polyphylla* | PQ221370 | |  | | Tierras Negras | Penjamo | Noviembre |
| 6-12 | *P. polyphylla* | PQ221371 | |  | | Tierras Negras | Penjamo | Noviembre |
| 6-13 | *P. polyphylla* | PQ221372 | |  | | Tierras Negras | Penjamo | Noviembre |
| 6-14 | *P. polyphylla* | PQ221373 | |  | | Tierras Negras | Penjamo | Noviembre |
| 6-16 | *P. polyphylla* | PQ221374 | | PQ246973 | | Tierras Negras | Penjamo | Noviembre |
| 6-17 | *P. polyphylla* | PQ221375 | |  | | Tierras Negras | Penjamo | Noviembre |
| 6-18 | *P. polyphylla* | PQ221376 | | PQ246974 | | Tierras Negras | Penjamo | Noviembre |
| 6-19 | *P. polyphylla* | PQ221377 | |  | | Tierras Negras | Penjamo | Noviembre |
| 6-22 | *P. polyphylla* | PQ221378 | |  | | Tierras Negras | Penjamo | Noviembre |
| 6-24 | *P. polyphylla* | PQ221379 | |  | | Tierras Negras | Penjamo | Noviembre |
| 6-27 | *P. polyphylla* | PQ221380 | |  | | Tierras Negras | Penjamo | Noviembre |
| 6-30 | *P. polyphylla* | PQ221381 | |  | | Tierras Negras | Penjamo | Noviembre |
| 7-13 | *P. polyphylla* | PQ221382 | |  | | Puruagua | Jerecuaro | Noviembre |
| 8-1 | *P. polyphylla* | PQ221383 | |  | | El Garbanzo | Irapuato | Diciembre |
| 8.2 | *P. polyphylla* |  | | PQ246975 | | El Garbanzo | Irapuato |  |
| 8-3 | *P. polyphylla* | PQ221384 | | PQ246976 | | El Garbanzo | Irapuato | Diciembre |
| 8-4 | *P. polyphylla* | PQ221385 | |  | | El Garbanzo | Irapuato | Diciembre |
| 8-6 | *P. polyphylla* | PQ221386 | | PQ246977 | | El Garbanzo | Irapuato | Diciembre |
| 8-7 | *P. polyphylla* | PQ221387 | |  | | El Garbanzo | Irapuato | Diciembre |
| 8-8 | *P. polyphylla* | PQ221388 | | PQ246978 | | El Garbanzo | Irapuato | Diciembre |
| 8-9 | *P. polyphylla* | PQ221389 | | PQ246979 | | El Garbanzo | Irapuato | Diciembre |
| 8-11 | *P. polyphylla* | PQ221390 | |  | | El Garbanzo | Irapuato | Diciembre |
| 8-12 | *P. polyphylla* | PQ221391 | | PQ246980 | | El Garbanzo | Irapuato | Diciembre |
| 8-13 | *P. polyphylla* | PQ221392 | |  | | El Garbanzo | Irapuato | Diciembre |
| 8-14 | *P. polyphylla* | PQ221393 | | PQ246981 | | El Garbanzo | Irapuato | Diciembre |
| 8-15 | *P. polyphylla* | PQ221394 | | PQ246982 | | El Garbanzo | Irapuato | Diciembre |
| 8-16 | *P. polyphylla* | PQ221395 | | PQ246983 | | El Garbanzo | Irapuato | Diciembre |
| 8-17 | *P. polyphylla* | PQ221396 | |  | | El Garbanzo | Irapuato | Diciembre |
| 8-18 | *P. polyphylla* | PQ221397 | | PQ246984 | | El Garbanzo | Irapuato | Diciembre |
| 8-19 | *P. polyphylla* | PQ221398 | | PQ246985 | | El Garbanzo | Irapuato | Diciembre |
| 8-20 | *P. polyphylla* | PQ221399 | |  | | El Garbanzo | Irapuato | Diciembre |
| 8-21 | *P. polyphylla* | PQ221400 | | PQ246986 | | El Garbanzo | Irapuato | Diciembre |
| 8-23 |  |  | | PQ246987 | |  |  |  |
| 8-24 |  |  | | PQ246988 | |  |  |  |
| 8-25 |  |  | | PQ246989 | |  |  |  |
| 9-1 | *P. polyphylla* | PQ221401 | |  | | Tierras Negras | Penjamo | Octubre |
| 9-2 | *P. polyphylla* | PQ221402 | |  | | Tierras Negras | Penjamo | Octubre |
| 9-3 | *P. polyphylla* | PQ221403 | |  | | Tierras Negras | Penjamo | Octubre |
| 9-5 | *P. polyphylla* | PQ221404 | | PQ246990 | | Tierras Negras | Penjamo | Octubre |
| 9-7 | *P. polyphylla* | PQ221405 | | PQ246991 | | Tierras Negras | Penjamo | Octubre |
| 9-8 | *P. polyphylla* | PQ221406 | | PQ246992 | | Tierras Negras | Penjamo | Octubre |
| 9-10 | *P. polyphylla* | PQ221407 | | PQ246993 | | Tierras Negras | Penjamo | Octubre |
| 9-13 | *P. polyphylla* | PQ221408 | |  | | Tierras Negras | Penjamo | Octubre |
| 9-19 | *P. polyphylla* | PQ221409 | |  | | Tierras Negras | Penjamo | Octubre |
| 9-20 | *P. polyphylla* | PQ221410 | |  | | Tierras Negras | Penjamo | Octubre |
| 9-23 | *P. polyphylla* |  | | PQ246994 | | Tierras Negras | Penjamo | Octubre |
| 9-25 | *P. polyphylla* |  | | PQ246995 | | Tierras Negras | Penjamo | Octubre |
| 10-1 | *P. polyphylla* | PQ221411 | |  | | El Garbanzo | Irapuato | Noviembre |
| 10-2 | *P. polyphylla* | PQ221412 | |  | | El Garbanzo | Irapuato | Noviembre |
| 10-3 | *P. polyphylla* | PQ221413 | | PQ246996 | | El Garbanzo | Irapuato | Noviembre |
| 10-4 | *P. polyphylla* | PQ221414 | | PQ246997 | | El Garbanzo | Irapuato | Noviembre |
| 10-5 | *P. polyphylla* | PQ221415 | | PQ246998 | | El Garbanzo | Irapuato | Noviembre |
| 10-6 | *P. polyphylla* | PQ221416 | |  | | El Garbanzo | Irapuato | Noviembre |
| 10-7 | *P. polyphylla* | PQ221417 | | PQ246999 | | El Garbanzo | Irapuato | Noviembre |
| 10-8 | *P. polyphylla* | PQ221418 | | PQ247000 | | El Garbanzo | Irapuato | Noviembre |
| 10-9 | *P. polyphylla* | PQ221419 | | PQ247001 | | El Garbanzo | Irapuato | Noviembre |
| 10-10 | *P. polyphylla* | PQ221420 | | PQ247002 | | El Garbanzo | Irapuato | Noviembre |
| 10-11 | *P. polyphylla* | PQ221421 | | PQ247003 | | El Garbanzo | Irapuato | Noviembre |
| 10-12 | *P. polyphylla* | PQ221422 | | PQ247004 | | El Garbanzo | Irapuato | Noviembre |
| 10-13 | *P. polyphylla* | PQ221423 | | PQ247005 | | El Garbanzo | Irapuato | Noviembre |
| 10-14 | *P. polyphylla* | PQ221424 | |  | | El Garbanzo | Irapuato | Noviembre |
| 10-16 | *P. polyphylla* | PQ221425 | |  | | El Garbanzo | Irapuato | Noviembre |
| 10-17 | *P. polyphylla* | PQ221426 | | PQ247006 | | El Garbanzo | Irapuato | Noviembre |
| 10-18 | *P. polyphylla* | PQ221427 | |  | | El Garbanzo | Irapuato | Noviembre |
| 10-19 | *P. polyphylla* | PQ221428 | | PQ247007 | | El Garbanzo | Irapuato | Noviembre |
| 10-20 | *P. polyphylla* | PQ221429 | | PQ247008 | | El Garbanzo | Irapuato | Noviembre |
| 10-21 | *P. polyphylla* | PQ221430 | |  | | El Garbanzo | Irapuato | Noviembre |
| 10-22 | *P. polyphylla* |  | | PQ247009 | | El Garbanzo | Irapuato | Noviembre |
| 3-7 | *Phyllophaga ravida* | PQ221431 | | PQ247010 | | Tierras Negras | Penjamo | Septiembre |
| 3-20 | *P. ravida* | PQ221432 | | PQ247011 | | Tierras Negras | Penjamo | Septiembre |
| 3-21 | *P. ravida* | PQ221433 | | PQ247012 | | Tierras Negras | Penjamo | Septiembre |
| 3-23 | *P. ravida* | PQ221434 | | PQ247013 | | Tierras Negras | Penjamo | Septiembre |
| 4-19 | *P. ravida* | PQ221435 | |  | | Puruagua | Jerecuaro | Septiembre |
| 4-26 | *P. ravida* | PQ221436 | |  | | Puruagua | Jerecuaro | Septiembre |
| 4-37 | *P. ravida* | PQ221437 | |  | | Puruagua | Jerecuaro | Septiembre |
| 6-6 | *P. ravida* | PQ221438 | |  | | Tierras Negras | Penjamo | Noviembre |
| 6-9 | *P. ravida* | PQ221439 | |  | | Tierras Negras | Penjamo | Noviembre |
| 6-21 | *P. ravida* | PQ221440 | | PQ247014 | | Tierras Negras | Penjamo | Noviembre |
| 6-26 | *P. ravida* | PQ221441 | | PQ247015 | | Tierras Negras | Penjamo | Noviembre |
| 9-11 | *P. ravida* | PQ221442 | | PQ247016 | | Tierras Negras | Penjamo | Octubre |
| 9-12 | *P. ravida* | PQ221443 | |  | | Tierras Negras | Penjamo | Octubre |
| 9-17 | *P. ravida* | PQ221444 | |  | | Tierras Negras | Penjamo | Octubre |
| 9-21 | *P. ravida* | PQ221445 | | PQ247017 | | Tierras Negras | Penjamo | Octubre |
| 3-2 | *Phyllophaga vetula* | PQ221446 | | PQ247018 | | Tierras Negras | Penjamo | Septiembre |
| 3-11 | *P. vetula* | PQ221447 | | PQ247019 | | Tierras Negras | Penjamo | Septiembre |
| 3-27 | *P. vetula* | PQ221448 | | PQ247020 | | Tierras Negras | Penjamo | Septiembre |
| 4-10 | *P. vetula* | PQ221449 | |  | | Puruagua | Jerecuaro | Septiembre |
| 4-36 | *P. vetula* | PQ221450 | | PQ247021 | | Puruagua | Jerecuaro | Septiembre |
| 6-7 | *P. vetula* | PQ221451 | |  | | Tierras Negras | Penjamo | Noviembre |
| 7-2 | *P. vetula* | PQ221452 | |  | | Puruagua | Jerecuaro | Noviembre |
| 8-5 | *P. vetula* | PQ221453 | |  | | El Garbanzo | Irapuato | Diciembre |
| 9-6 | *P. vetula* | PQ221454 | | PQ247022 | | Tierras Negras | Penjamo | Octubre |
| 9-16 | *P. vetula* | PQ221455 | | PQ247023 | | Tierras Negras | Penjamo | Octubre |
| 9-18 | *P. vetula* | PQ221456 | | PQ247024 | | Tierras Negras | Penjamo | Octubre |
